# Identification and validation of major QTLs associated with low seed coat deficiency of natto soybean seeds (*Glycine max* L.)

**DOI:** 10.1007/s00122-020-03662-5

**Published:** 2020-08-26

**Authors:** Qian Zhu, Diana M. Escamilla, Xingbo Wu, Qijian Song, Song Li, M. Luciana Rosso, Nilanka Lord, Futi Xie, Bo Zhang

**Affiliations:** 1 School of Plant and Environmental Sciences, Virginia Tech, Blacksburg, VA 24061 USA; 2 College of Agronomy, Shenyang Agricultural University, Shenyang, 110866 People’s Republic of China; 3 Department of Agronomy, Purdue University, West Lafayette, IN 47907 USA; 4 Soybean Genomics and Improvement Laboratory, Beltsville Agricultural Research Center, USDA-ARS, Beltsville, MD 20705 USA

## Abstract

**Key message:**

Two major QTLs associated with low seed coat deficiency of soybean seeds were identified in two biparental populations, and three SNP markers were validated to assist low-SCD natto soybean breeding selection.

**Abstract:**

Soybean seed coat deficiency (SCD), known as seed coat cracking during soaking in the natto production process, is problematic because split or broken beans clog production lines and increases production costs. Development of natto soybean cultivars with low SCD is crucial to support the growth of the natto industry. Unfortunately, information on the genetic control of SCD in soybean, which is desperately needed to facilitate breeding selection, remains sparse. In this study, two F_2_ populations derived from V11-0883 × V12-1626 (Pop 1) and V11-0883 × V12-1885 (Pop 2) were developed and genotyped with BARCSoySNP6K Beadchips and F_2_-derived lines were evaluated for SCD in three consecutive years (2016–2018) in order to identify quantitative trait loci (QTLs) associated with low SCD in soybean. A total of 17 QTLs underlying SCD were identified in two populations. Among these, two major and stable QTLs, *qSCD15* on chromosome 15 and *qSCD20* on chromosome 20, were detected across multiple years. These QTLs explained up to 30.3% of the phenotypic variation for SCD in Pop 1 and 6.1% in Pop 2 across years. Three SNP markers associated with the *qSCD20* were validated in additional four biparental populations. The average selection efficiency of low-SCD soybean was 77% based on two tightly linked markers, Gm20_34626867 and Gm20_34942502, and 64% based on the marker Gm20_35625615. The novel and stable QTLs identified in this study will facilitate elucidation of the genetic mechanism controlling SCD in soybean, and the markers will significantly accelerate breeding for low-SCD soybean through marker-assisted selection.

**Electronic supplementary material:**

The online version of this article (10.1007/s00122-020-03662-5) contains supplementary material, which is available to authorized users.

## Introduction

**Fig. 1:**
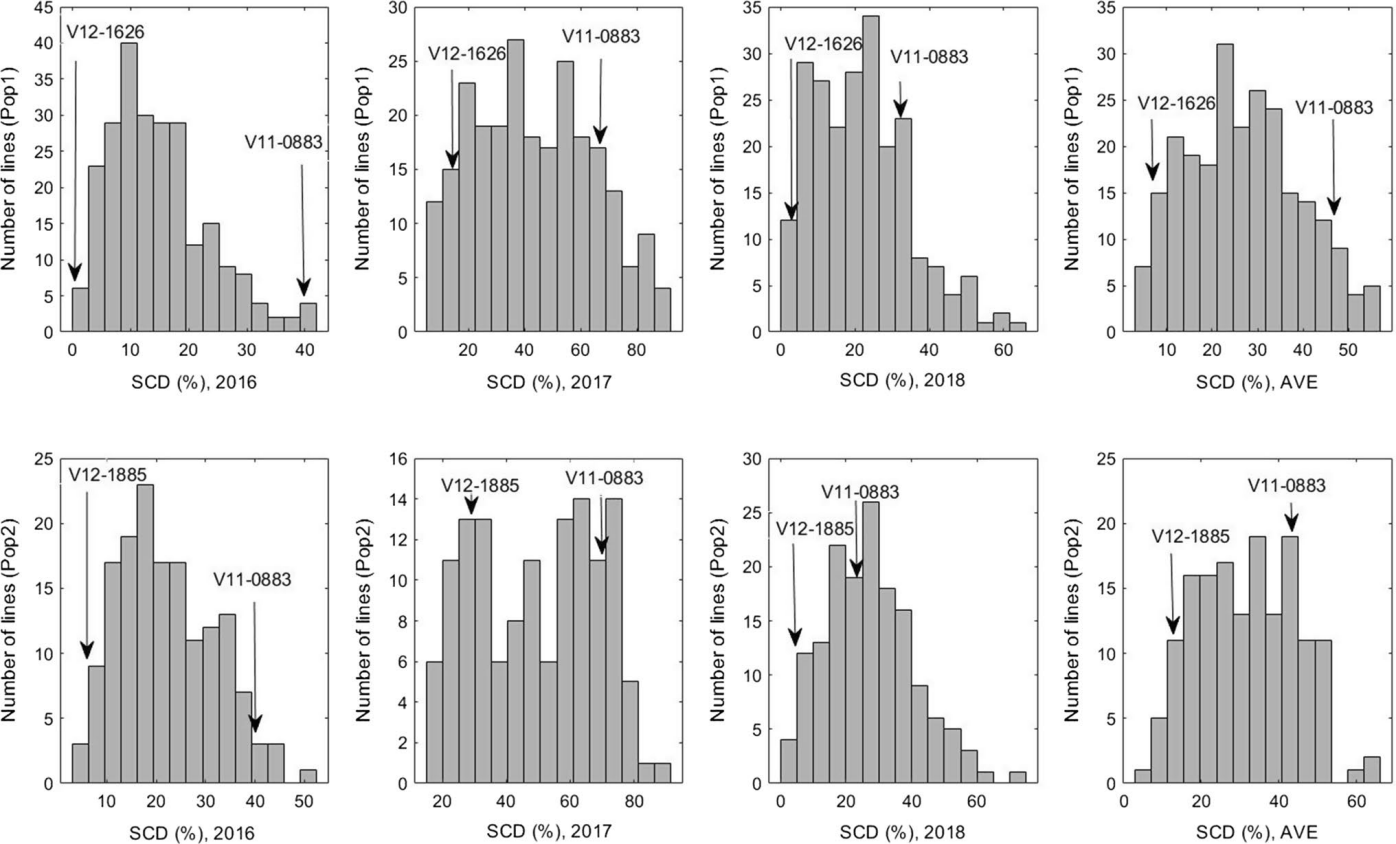
Distribution of seed coat deficiency (SCD) in 3 years (2016, 2017 and 2018) and their mean overall years (AVE) of Pop 1 (V11-0883 × V12-1626) and Pop 2 (V11-0883 × V12-1885)

Because of its high content of protein, fiber, amino acids, and isoflavones, soybean (*Glycine max* L. Merr.) has become increasingly appealing for human consumption as a nutritional and functional food (Gibbs et al. 2004; Ikeda et al. 2006; Sanjukta and Rai 2016). Natto, fermented whole soybeans, is a popular soyfood product in Japan and is well known for its nutrition, unique flavor and stickiness (Hu et al. 2010; Wei and Chang 2004). The USA has been the largest exporter of natto-type soybeans to Japan since the early 1990s. Utilization of US soybeans for natto production in Japan has been predicated on the ability of domestic growers to continuously meet the needs of natto manufacturers by supplying them with value-added, food-grade soybean seeds that result in high quality natto products (Ikeda et al. 2006; Yoshikawa et al. 2014). Soybean seeds must meet several quality standards for natto production: seeds must be small (< 9 g/100 seeds), have high water absorption capacity (Cook and Rainey 2010), be uniform in size, have minimum change in constituents during storage, be round in shape, and have clear hilum and yellow and smooth seed coat (Cui et al. 2004; Geater et al. 2000; Hosoi and Kiuchi 2003). Seeds with cracked seed coats or seed discoloration, or excessively flat seeds, were unqualified for natto production (Montague Farms, Inc., Center Cross, VA, personal communication). Thus, the quality of natto products is mainly determined by soybean cultivars, processing conditions (soaking, cooking and fermentation), and bacteria strains (Wei and Chang 2004). The initial water absorption in the natto producing process, where seeds are softened and soluble sugars are released, is an important step before natto fermentation (Cook and Rainey 2010). During this process, the seed coat regulates water absorption and prevents the destruction of seed tissue at the beginning of seed imbibition (Copeland and McDonald 2001; Koizumi et al. 2008). It is ideal for natto soybean to keep the seed coat intact despite weakening and expanding during absorption. However, inferior seed coat integrity has been frequently observed in USA, which was mainly caused by the seed coat cracking during water absorption and defined as seed coat deficiency (SCD) (Fig. 1S) (Cook and Rainey 2010 and personal communications). SCD is an undesirable trait for natto soybeans because it affects final natto appearance and clogs production lines which increases production costs and reduces profit (Yasui et al. 2017). The phenotypic data of SCD indicated that breeding selection is feasible to efficiently reduce SCD (Cook and Rainey 2010), but it is challenging due to the time-consuming, subjective, and laborious nature of phenotyping and the poorly understood influence of environment (Escamilla et al. 2019). Marker-assisted selection (MAS) has been incorporated into most soybean breeding programs (He et al. 2004); however, it has not been implanted in the low-SCD soybean selection due to limited genetic control information of this SCD trait. A recent study identified eight QTLs located on chromosomes 4, 6 and 8 that are associated with seed coat cracking after soaking and cooking (Yasui et al. 2017), but none of these QTLs were validated for MAS in breeding selection. SoyBase (http://www.soybase.org/ accessed Aug. 26, 2019) also reported 14 QTLs related to seed coat cracking in dry seeds; however, the phenotype of those QTLs was obtained by determining physical seed coat cracking after harvest, which was physiologically different compared to seed coat deficiency in natto soybean production. Currently, there is a widespread lack of available molecular tools that can be used for MAS of the low-SCD trait in soybean.

Understanding of the genetic control of SCD is essential for development of molecular tools that can be used to improve breeding selection for low-SCD natto soybean cultivars. Therefore, the objectives of this study were to (1) identify QTLs associated with seed coat deficiency in soybean using two populations across multiple environments, and (2) validate these QTLs through Kompetitive Allele Specific PCR (KASP) assays using 86 breeding lines from four validation populations.

## Materials and methods

### Population development and experiment design

**Fig. 2:**
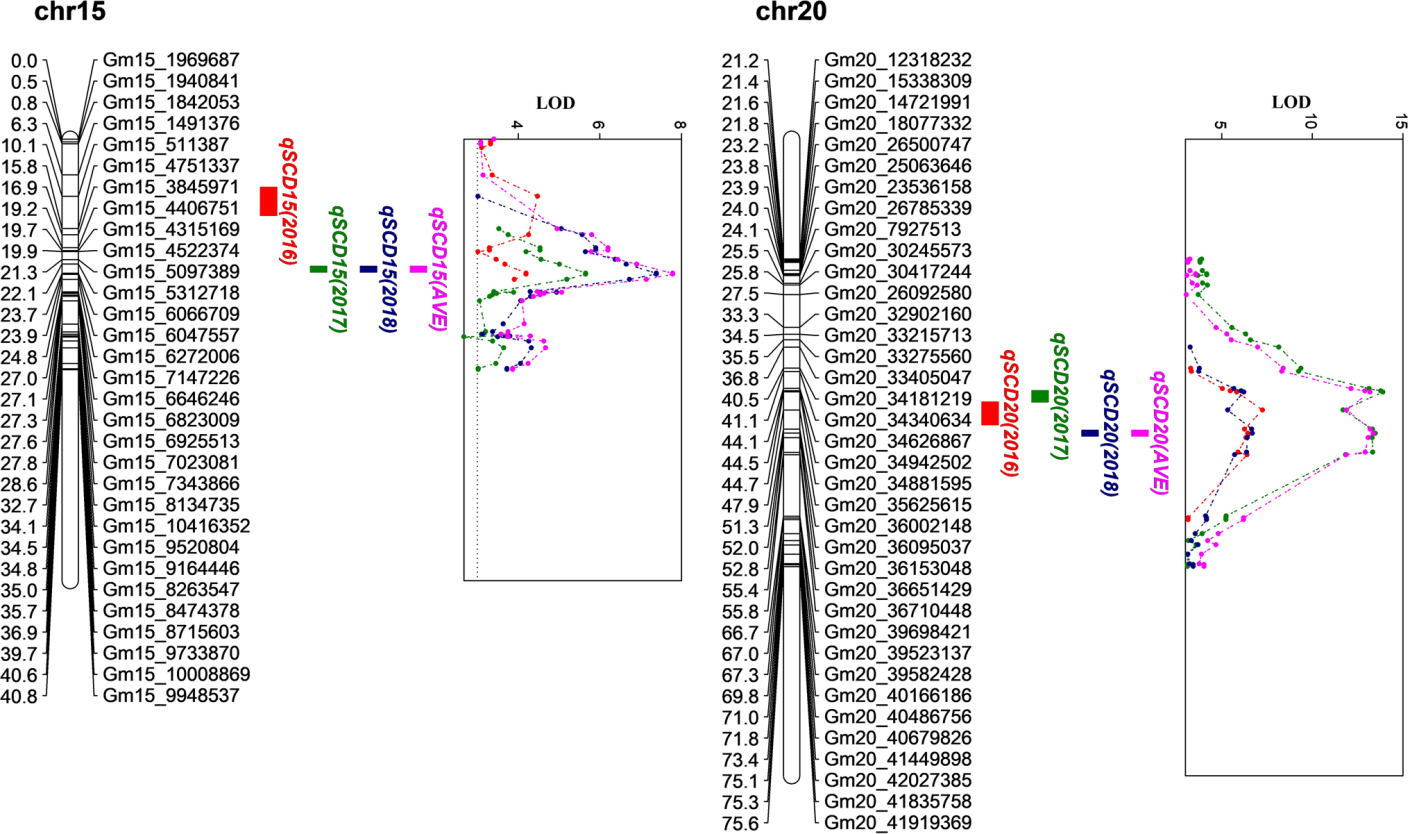
Mapping for seed coat deficiency (SCD) in the Pop 1 (V11-0883 × V12-1626) in individual and across years (AVE). QTL nomenclature is in the form of *qTraitChr.* Colored intervals refer to the mapped QTLs detected by CIM and/or IM in different environments, the curves indicate the physical position of markers against LOD score detected on chromosomes, and lines with different colors represent different environments

Two populations were developed by crossing small-seeded soybean breeding lines V11-0883 × V12-1626 (Pop 1) and V11-0883 × V12-1885 (Pop 2) (Pedigree was shown in Fig. 2S). The female parent V11-0883 produces the high-SCD phenotype, while the two male parents, V12-1626 and V12-1885, produce the low-SCD phenotype. Crosses were made in Blacksburg, VA in 2014, and the F_1_ generation was planted at the same location the following year. Six SSR markers (Satt449, Satt197, Satt281, Satt268, Satt431 and Satt345), which were polymorphic between parents, were used to verify true hybrids. A total of 240 and 153 F_2_ individuals from Pop 1 and Pop 2, respectively, were advanced to F_3_ at a winter nursery during the winter of 2015. In 2016, the F_2:3_ lines from two populations were separately spaced planted (0.03 m) in single, 3.05-mm-long rows with 0.76 m row spacing (with a seeding rate of 70,542 plants per hectare) arranged in a complete randomized design with three replications in Blacksburg, VA. F_2:4_ and F_2:5_ lines were harvested and replanted in subsequent years at the same growing location using the same experimental design.

In all years, fertilizer was applied according to soil test recommendations and pre-emergent herbicide (Dual Magnum) was applied at rate of 2 L ha^−1^ to reduce weed pressure. No irrigation or insecticides was applied. Rows were inspected for flower and pubescence color each year in order to control population purity and avoid contamination. Seeds were harvested 5–10 days after 95% of the plants in a row reached R8 maturity.

### Determination of seed coat deficiency

Seeds were stored in the seed storage room until seed moisture stabilized between 10 and 12.2% (Cook and Rainey 2010). Seeds with cracked seed coat or seed discoloration, or excessively flat seeds, were removed. One hundred intact soybean seeds of each plot were subsampled for SCD determination. A modified method from protocols described in previous studies (Cook and Rainey 2010; Rodda et al. 1973) was used for easy observation. Briefly, a 100-seed sample was placed in a plastic container with 50 ml of 1% commercial bleach solution for ten minutes; after soaking, the samples were drained and scored for SCD. Seeds that showed cracking were severely blistered around the hilum, or whose seed coats had detached from the hull were considered seed coat deficient (Fig. 1S) (Cook and Rainey 2010). The percentage of seed coat deficient seeds was used to score SCD.

### Statistical data analysis

The SPSS statistical version 20.0 (SPSS Inc., Chicago, USA) was used to summarize the descriptive statistics of the SCD for each population. Normality assumption was assessed by the Shapiro–Wilk test and normal probability plots. Analysis of variance (ANOVA) was used to evaluate variation of SCD within and between each environment in each population. Histograms of SCD distributions were elaborated by R function “*hist()*”. The variance components were calculated and used to estimate the broad-sense heritability using the following equation:

$$H^{2} = \, s^{2}_{\text{g}} / \, \left[ { \, s^{2}_{\text{g}} + \, \left( {s^{2}_{\text{ge}} /e} \right) \, + \, \left( {s^{2} /re} \right)} \right]$$

where *H*^2^ is heritability, $s^{ 2}_{\text{g}}$ is genotypic variance, $s^{ 2}_{\text{ge}}$ is genotype × environment interaction variance, *s*^2^ is error variance, *r* is the number of replications, and *e* is the number of environments (Nyquist and Baker 1991).

### DNA extraction and genotyping

Leaf tissue samples from F_2_ individuals and parental lines were collected from the field in centrifuge tubes and stored at −80 °C until extraction. For genomic DNA extraction, leaf tissue samples were freeze-dried at − 0.220 mbar with the collector temperature set at − 56 °C (FreeZone 6 Dryer system, Labconco, Kansas City, MO, USA). Lyophilized tissues were ground in liquid nitrogen using glass stirring rods. Total genomic DNA of each sample was isolated following a modified CTAB method (Saghai-Maroof et al. 1984).

Fifty nanograms of genomic DNA for each genotype were sent to USDA–ARS Soybean Genomics and Improvement Laboratory (Beltsville, MD) for genotyping using the Illumina 6000-SNP BARCSoySNP6K Beadchip, selected from the SoySNP50K (Song et al. 2013). The SNP allele calling was conducted in GenomeStudio Module v2.0.3 (Illumina, Inc.). The low seed coat deficiency parents were scored as A, and high seed coat deficiency parents were scored as B. SNPs with no call and the monomorphic SNPs between parents were discarded. SNPs with low minor allele frequency (MAF) (< 10%) and high missing data ratio (< 5%), as well as severe segregation distortion, were filtered for quality control.

### Linkage map construction and QTL analysis

Linkage maps were constructed by Joinmap 4.0 (Van Ooijen 2006) using a regression approach with a minimum logarithm of odds (LOD) threshold of 3. Recombination frequencies were converted to centimorgan (cM) using Kosambi mapping function (Kosambi 1943). To ensure consistency, QTL analyses were performed by single-marker analysis (SMA), interval mapping (IM) and composite interval mapping (CIM) implemented in ICiMapping v 4.1 (Wang et al. 2016). For SMA, *p* < 0.0001 was used as the experiment wide threshold for significant markers. In the CIM and IM, the experiment wide threshold was determined by 1000 permutation at significance level of 0.05 with a walk speed of 1 cM. MapChart (Voorrips 2002) was used to create the LOD plots based on JoinMap 4.0 and ICiMapping v 4.1 results.

### KASP marker development

The SNPs tightly linked to major QTL identified in the mapping populations were converted into Kompetitive Allele Specific PCR (KASP) SNP genotyping assays (LGC, Middlesex, UK) with the flanking sequences obtained from the *G. max* genome Glyma.Wm82.a1 (Schmutz et al. 2010). The KASP oligos were synthesized by Integrated DNA Technologies (IDT, Iowa, USA), with primers carrying FAM tail (5′-GAAGGTGACCAAGTTCATGCT-3′) or VIC tail (5′-GAAGGTCGGAGTCAACGGATT-3′), and the target SNP in the 3′ end. Primer mix and PCR reaction were set up following LGC Genomics recommendation (46 µL distilled water, 30 µL common primer [100 µM], and 12 µL of each tailed primer [100 µM]). Thermocycling conditions consisted of the initial hot-start step at 95 °C for 15 min, followed by 10 cycles of touchdown PCR (annealing 65 °C to 57 °C, decreasing 0.8 °C per cycle), then 35 cycles of 20 s at 94 °C and 60 s at 57 °C. PCR and fluorescent endpoint reading were performed in FLUOstar Omega microplate reader (BMG LABTECH).

### KASP marker validation

A total of 86 breeding lines from four biparental populations (MFS-561 × V09-0579, MFS-561 × V09-3876, V05-5973W × V09-3876, V05-5973W × V09-3984) were planted in Warsaw, VA for marker validation. The female parents, MFS-561 and V05-5973W produce the low-SCD phenotype, while all male lines produce the high-SCD phenotype. SCD was determined by the same method as in the mapping populations. Breeding lines with SCD less than or equal to that of either male parent were considered low-SCD lines. Selection efficiency (SE) of the selected markers linked to low SCD was calculated as follows: SE = (NC/NS) × 100, where NC is the number of low-SCD lines selected correctly by marker and NS is the total number of lines selected as low SCD by marker.

## Results

### Phenotypic analysis of seed coat deficiency

Both populations and their parental lines were scored for SCD during 2016–2018. In both populations, the SCD of the female parent V11-0883 (45.6%) exceeded that of both male parents (V12-1626, 7.3% and V12-1885, 13.7%) in all 3 years (Table 1). Among eight tests (two populations in 3 years plus mean across 3 years), three (SCD in 2017 and mean for Pop1; SCD in 2018 for Pop2) showed normal distribution (K–S test, *p *>0.2), while the rest showed continuous distribution (Table 1, Fig. 1), suggesting SCD was inherited as a quantitative trait. Large SCD variation was observed among individuals in both populations across all 3 years. For both populations, a larger degree of variation was observed in 2017 than in 2016 and 2018. The skewness was positive for SCD in all years and populations with the exception of Pop 2 in 2017, which displayed asymmetry toward the low-SCD end of the curve. Significant differences (*p *≤ 0.0001) were detected when comparing SCD among genotypes, years, and the interaction between genotype and year in both populations (Table 1). The broad-sense heritability (*H*^2^) of SCD was 0.67 and 0.83 for Pop 1 and Pop 2, respectively, with an overall mean of 0.75.

**Table 1:** Descriptive statistics for seed coat deficiency (SCD) of two populations in 2016, 2017 and 2018

Population^a^	Year	Max (%)^b^	Min (%)^c^	Mean (%)	SE^d^	Skewness	Kurtosis	*p* value	P1^e^	P2^f^	P3^g^	*H*^2h^	K–S test^i^
Pop 1	2016*	48.0	0	14.8	0.39	0.88	0.52	< 0.0001	40.0	0.5	–	–	*p *< 0.001
	2017*	94.0	3.0	43.3	1.02	0.18	− 0.83	< 0.0001	68.0	18.5	–	–	*p *>0.2
	2018*	70.0	0	21.7	0.66	0.71	0.31	< 0.0001	33.0	3.0	–	–	*p *= 0.004
	2016/2017/2018**	55.8	4.5	26.8	0.46	0.24	− 0.69	< 0.0001	47.0	7.3	–	0.67	*p *>0.2
Pop 2	2016*	59.0	2.0	22.0	0.78	0.43	− 0.36	0.0133	40.0	–	6.5	–	*p *= 0.017
	2017*	96.0	6.0	49.3	1.98	− 0.07	− 1.23	< 0.0001	68.0	–	29.0	–	*p *= 0.001
	2018*	75.0	2.0	27.0	1.10	0.56	0.24	0.0132	24.5	–	5.5	–	*p *>0.2
	2016/2017/2018**	65.0	5.3	31.6	1.02	0.11	− 0.72	0.0111	44.2	–	13.7	0.83	*p *= 0.039

^a^Pop 1 and Pop 2 were developed from the crosses of V11-0883 × V12-1626 and V11-0883 × V12-1885, respectively

^b^Max: maximum

^c^Min: minimum

^d^SE: standard error

^e^P1: V11-0883

^f^P2: V12-1626

^g^P3: V12-1885

^h^*H*^2^: Broad-sense heritability in combined environments (2016,2017, and 2018)

^i^K–S test: Kolmogorov–Smirnov test for normality distribution, probability was shown

*Significant difference among genotypes at the *p* < 0.0001

**Significant difference among genotypes (G), years (Y) and G × Y interaction at the *p* < 0.0001

### QTL associated with SCD

Out of 6000 SNPs, 1318 and 1637 SNPs were polymorphic between the parental lines and subsequently selected after date filtration for linkage map construction in Pop 1 and Pop 2, respectively. A total of 1258 SNPs were mapped to 20 chromosomes (Chr.) in Pop 1 with a total genetic distance of 1826 cM ranging from 44.9 cM (Chr. 16) to 128.3 cM (Chr. 3) with an average length of 91.3 cM (Table 1S). The average distance between adjacent markers in Pop 1 was 1.6 cM. For Pop 2, a total of 1604 SNPs were mapped to 20 chromosomes (Table 2S). The total genetic length of LGs for Pop 2 was 1189 cM, with Chr. 10 being the longest (90.5 cM) and Chr. 16 being the shortest (33.3 cM). The average length of LG was 59.3 cM with an average marker interval of 0.8 cM.

**Fig. 3:**
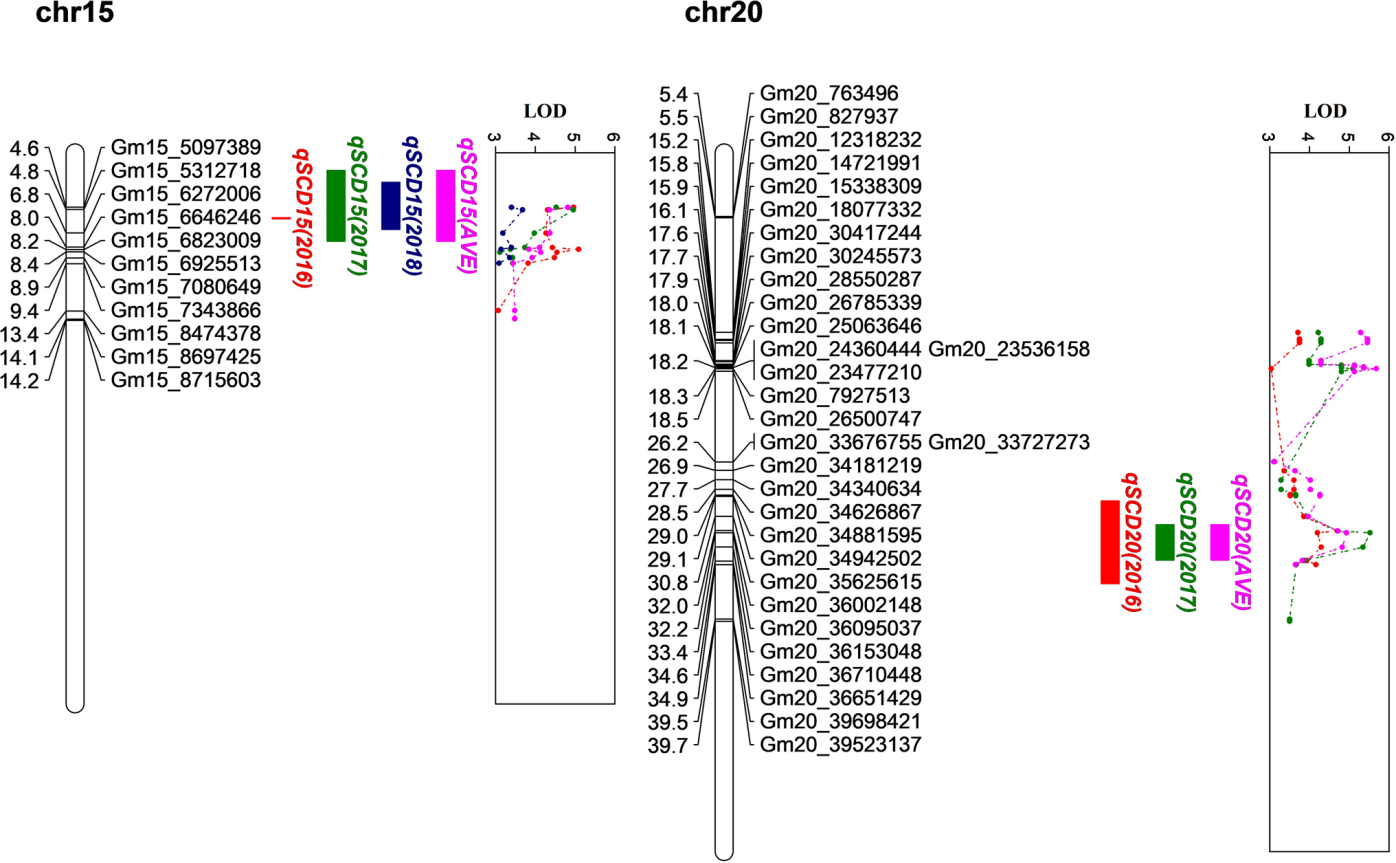
Mapping for seed coat deficiency (SCD) in the Pop 2 (V11-0883 × V12-1885) in individual and across years (AVE). QTL nomenclature is in the form of *qTraitChr.* Colored intervals refer to the mapped QTLs detected by CIM and/or IM in different environments; the curves indicate the physical position of markers against LOD score detected on chromosomes, and lines with different colors represent different environments

Genome-wide QTL analysis was performed based on the genetic map and phenotypic data of each population using composite interval mapping (CIM) and interval mapping (IM) in each year, as well as across 3 years. In Pop 1, two major QTLs were detected on Chr. 15 and Chr. 20 (Table 2, Fig. 2), while six other QTLs were detected on Chr. 3, 5, 6, 7, 10 and 14. The two stable QTLs, namely *qSCD15* and *qSCD20*, were detected in each year and across 3 years, explaining 5.3–23.8% phenotypic variation. Meanwhile, *qSCD15* and *qSCD20* were also repeatedly detected in Pop 2 (Table 2, Fig. 3), along with seven other QTLs distributed on Chr. 1, 13, 16, 18 and 19. Among the two stable QTLs detected in Pop 2, *qSCD15* was detected in individual and across years, explaining 3.1–10.4% phenotypic variation, while *qSCD20* were detected in 2016, 2017 and across years, explaining 3.0–13.8% phenotypic variation. The additive effect of all detected QTLs was negative, indicating the male parents (V12-1626 and V12-1885) contributed negative allele decreasing SCD, while the female parent (V11-0883) contributed positive allele increasing SCD.

**Table 2:** Quantitative trait loci for seed coat deficiency (SCD) in the mapping populations

Pop^a^	Env^b^	QTL name^c^	Position (cM)	Chr^d^	Flanking markers^e^	GI (cM)^f^	Wm82.a2.v1 Physical interval (bp)^g^	LOD^h^	PVE(%)^i^	Add^j^	Method^k^
Pop 1	2016	*qSCD14*	28	14	Gm14_6024101-Gm14_4956317	26.5–29.5	6,135,158–5,040,727	3.4	4.6	− 1.9	CIM
		***qSCD15***	12	15	Gm15_511387-Gm15_4751337	8.5–13.5	511,864–4,770,814	4.5 − 6.1	**6.6 **− 10.1	− 2.8 to − 2.9	CIM, IM, SMA
		***qSCD20***	48	20	Gm20_35625615-Gm20_36002148	46.5–50.5	36,720,824–37,097,315	7.3 − 8.4	**7.5 **− 11.3	− 3.0 to − 3.1	CIM, IM, SMA
	2017	*qSCD3*	4	3	Gm03_241447-Gm03_715393	0–8.5	239,949–714,448	4.5 − 7.2	4.2 − 5.3	− 4.4 to − 6.1	CIM, IM, SMA
		*qSCD10*	25	10	Gm10_11518881-Gm10_26188429	22.5–25.5	11,710,604–16,707,334	3.4 − 4.5	4.1 − 5.0	− 4.3 to − 6.1	CIM, IM, SMA
		***qSCD15***	23	15	Gm15_5312718-Gm15_6066709	22.5–23.5	5,331,364–6,085,794	5.0 − 5.7	5.3 − **7.0**	− 5.0 to − 6.9	CIM, IM, SMA
		***qSCD20***	45	20	Gm20_34881595-Gm20_35625615	44.5–46.5	36,021,058–36,720,824	14.1 − 15.2	12.2 − **23.8**	− 9.3 to − 10.4	CIM, IM, SMA
	2018	*qSCD5*	98	5	Gm05_36671535-Gm05_41740936	95.5–98	36,962,030–38,597,425	3.1	4.9	− 2.7	CIM
		*qSCD6*	51	6	Gm06_17258654-Gm06_38499465	46.5–56.5	17,303,937–39,188,086	5.5 − 5.9	9.3 − 9.5	− 3.6 to − 4.6	CIM, IM, SMA
		*qSCD7*	72	7	Gm07_17030454-Gm07_16979586	71.5–72.5	17,116,283–17,065,562	3.1	4.8	− 2.6	CIM
		***qSCD15***	23	15	Gm15_5312718-Gm15_6066709	22.5–23.5	5,331,364–6,085,794	6.3 − 7.4	10.2 − 10.4	− 3.7 to − 4.9	CIM, IM, SMA
		***qSCD20***	52	20	Gm20_36002148-Gm20_36095037	51.5–52.5	37,097,315–37,190,252	6.1 − 6.7	9.4 − 9.7	− 3.7 to − 4.7	CIM, IM, SMA
	AVE	*qSCD6*	51	6	Gm06_17258654-Gm06_38499465	46.5–56.5	17,303,937–39,188,086	5.2 − 7.3	7.3 − 7.8	− 3.0 to − 4.7	CIM, IM, SMA
		*qSCD10*	26	10	Gm10_23967467-Gm10_12080275	22.5–27.5	20,275,514–12,272,454	3.1 − 3.8	3.8	− 3.3	IM, SMA
		*qSCD14*	30	14	Gm14_5603904-Gm14_5655487	29.5–30.5	5,714,996–5,766,579	2.9	3.5	− 2.1	CIM
		***qSCD15***	23	15	Gm15_5312718-Gm15_6066709	22.5–23.5	5,331,364–6,085,794	7.3 − 7.8	7.5 − 9.5	− 3.5 to − 4.6	CIM, IM, SMA
		***qSCD20***	52	20	Gm20_36002148-Gm20_36095037	51.5–52.5	37,097,315–37,190,252	13.7 − 15.1	12.9 − 20.8	− 5.1 to − 6.0	CIM, IM, SMA
Pop 2	2016	*qSCD1_1*	15	1	Gm01_1887205-Gm01_49322760	13.5–16.5	1,887,609–50,206,347	4.6	5.3	− 4.3	IM, SMA
		*qSCD1_2*	25	1	Gm01_1653315-Gm01_50262496	24.5–26.5	1,653,600–51,147,675	5.4 − 7.2	6.8 − 17.4	− 3.7 to − 5.2	CIM, IM, SMA
		*qSCD1_3*	50	1	Gm01_31906055-Gm01_7052156	46.5–55.5	33,203,133–7,071,117	4.9	4.1	− 3.9	IM, SMA
		*qSCD13*	21	13	Gm13_29677928-Gm13_28206014	20.37–22.37	30,875,555–29,399,384	5.5	4.3	− 3.8	IM, SMA
		***qSCD15***	6	15	Gm15_5312718-Gm15_6272006	5.5–6.5	5,331,364–6,291,081	4.8	3.9	− 4.1	IM, SMA
		*qSCD18_1*	21	18	Gm18_15324039-Gm18_7348168	20.5–21.5	15,066,204–7,370,390	4.1	3.3	− 3.5	IM, SMA
		***qSCD20***	32	20	Gm20_35625615-Gm20_36002148	29.5–36.5	36,720,824–37,097,315	4.7	3.6	− 3.7	IM, SMA
	2017	*qSCD1_3*	51	1	Gm01_31906055-Gm01_7052156	48.5–55.5	33,203,133–7,071,117	4.0 − 5.5	4.8 − 14.0	− 6.7 to − 8.6	CIM, IM, SMA
		***qSCD15***	5	15	Gm15_5312718-Gm15_6272006	1.5–7.5	5,331,364–6,291,081	4.9	**3.9**	− 8.3	IM, SMA
		*qSCD16*	2	16	Gm16_103052-Gm16_1755870	0–7.5	102,882–1,772,720	4.2	3.6	− 7.5	IM, SMA
		*qSCD18_2*	5	18	Gm18_122382-Gm18_1308443	0–5.5	122,380–1,308,798	5.5	4.9	− 8.5	IM, SMA
		*qSCD19*	67	19	Gm19_41918030-Gm19_50486916	65.5–68.5	42,120,908–50,607,336	5.3	5.8	− 9.5	IM, SMA
		*qSCD20_2*	18	20	Gm20_26785339-Gm20_25063646	15.5–21.5	27,947,431–26,214,468	4.8	3.8	− 8.0	IM, SMA
		***qSCD20***	33	20	Gm20_36095037-Gm20_36153048	31.5–34.5	37,190,252–37,248,263	4.1 − 5.4	4.3 − **13.8**	− 6.8 to − 8.4	CIM, IM, SMA
	2018	***qSCD15***	5	15	Gm15_5312718-Gm15_6272006	2.5–6.5	5,331,364–6,291,081	3.8	10.4	− 5.1	CIM, IM, SMA
	AVE	*qSCD1_2*	25	1	Gm01_1653315-Gm01_50262496	24.5–26.5	1,653,600–51,147,675	3.6	3.1	− 5.0	IM, SMA
		*qSCD1_3*	50	1	Gm01_31906055-Gm01_7052156	46.5–55.5	33,203,133–7,071,117	4.2 − 6.0	4.1 − 15.5	− 4.5 to − 5.6	CIM, IM, SMA
		***qSCD15***	5	15	Gm15_5312718-Gm15_6272006	2.5–6.5	5,331,364–6,291,081	4.9	3.1	− 5.3	IM, SMA
		*qSCD20_2*	12	20	Gm20_827937-Gm20_12318232	7.5–14.5	824,049–8,318,718	5.7	4.3	− 5.8	IM, SMA
		***qSCD20***	33	20	Gm20_36095037-Gm20_36153048	31.5–34.5	37,190,252–37,248,263	4.9	3.0	− 5.0	IM, SMA

Bold letters or values are QTLs names or the least or the maximum percentage of variance explained by corresponding QTL, respectively, mentioned in the text

^**a**^Pop 1 and Pop 2 were developed from the crosses of V11-0883 × V12-1626 and V11-0883 × V12-1885, respectively

^**b**^Env: the individual years and across years (AVE) in which the QTL was detected

^**c**^The name of each QTL is a composite of SCD followed by the chromosome number, QTLs detected in different methods with the same or overlapping maker interval were designated as on QTL

^d^Chr: chromosome

^e^Flanking markers: the markers flanking the QTL

^f^GI: genetic interval

^g^Physical interval according to genome position at Wm82.a2.v

^h^LOD: logarithm of odds, data showed the range of the values detected across methods

^i^PVE: percentage of variance explained, data showed the range of the values detected across methods

^j^Add: additive effect, data showed the range of the values detected across methods

^k^Methods: by which the QTL was detected, data presented were the range of different methods

The two stable QTLs, *qSCD15* and *qSCD20*, were identified in both populations across multiple years and could together explain 21.4–30.8% and 6.3% of phenotypic variation for Pop 1 and Pop 2, respectively. The high stability and explained phenotypic variation percentage suggest that *qSCD15* and *qSCD20* are major QTLs for SCD in soybeans. Furthermore, the single-marker analysis (SMA) confirmed the significant associations between SCD and eleven markers within the two major QTL regions identified in Pop 1 (Table 3). Two markers within the region of *qSCD15* (Gm15_5312718 and Gm15_6272006) were detected in 2018 and across years, and nine markers from the *qSCD20* region were detected in 2017 and across years.

**Table 3:** SNPs highly significant associated with seed coat deficiency (SCD) detected by single-marker analysis (SMA) in Pop1 (V11-0883 × V12-1626) within two stable QTL regions

QTL^a^	Marker ID	Position (cM)	LOD^b^			
			2016	2017	2018	AVE
***qSCD15***	Gm15_5312718	22.1	–	–	6.7*	5.9*
	Gm15_6272006	24.8	–	–	6.7*	6.0*
***qSCD20***	Gm20_34626867	44.1	–	13.1*	–	12.1*
	Gm20_34942502	44.5	–	13.8*	–	12.8*
	Gm20_34881595	44.7	–	13.9*	–	13.2*
	Gm20_35625615	47.9	7.3*	11.7*	–	11.8*
	Gm20_36002148	51.3	6.3*	13.3*	6.7*	12.9*
	Gm20_36095037	52.0	6.4*	13.5*	6.7*	13.1*
	Gm20_36153048	52.8	6.3*	13.3*	6.4*	13.1*
	Gm20_36651429	55.4	–	13.3*	6.4*	12.9*
	Gm20_36710448	55.8	6.4*	11.8*	–	11.9*

^a^QTL: the QTL detected by CIM and/or IM, *see* in Table 2

^b^LOD: logarithm of odds

*SNPs significantly associated with seed coat deficiency at p < 0.0001

### SNP validation via KASP genotyping assay

Eleven SNPs from the two stable QTLs (*qSCD15* and *qSCD20*) that showed tight linkage to SCD (*p *< 0.001) were selected for marker validation (Table 3). None of the SNPs derived from the QTLs located on Chr. 15 segregated in the parental lines of the validation populations. In addition, three SNPs (Gm20_34626867, Gm20_34942502 and Gm20_35625615) located on Chr. 20 were found to be polymorphic between the parental lines of the validation populations. Gm20_34626867 and Gm20_34942502, located only 0.39 cM apart, had high selection efficiency (83% and 100%, respectively) in MFS-561-derived populations, and low selection efficiency (25% and 50%) in V05-5973 W-derived populations (Table 4). The other marker, Gm20_35625615, located 3.4 cM away from Gm20_34942502, showed variable selection efficiency across all four populations (57%, 92%, 33% or 50%). The combined average selection efficiency of marker Gm20_34626867 and Gm20_34942502 across all four validation populations was 77%, which exceeded that of marker Gm20_35625615 (64%) by just over 12%.

**Table 4:** Markers selection efficiency (SE) in four validation populations developed in crosses using one low-SCD soybean in each combination and three SNPs significantly associated with SCD

Validation population	Line no.	Gm20_34626867			Gm20_34942502			Gm20_35625615		
		NC^a^	NS^b^	SE (%)	NC^a^	NS^b^	SE (%)	NC^a^	NS^b^	SE (%)
MFS-561 × V09-0579	34	5	6	83	5	6	83	12	21	57
MFS-561 × V09-3876	23	12	12	100	12	12	100	11	12	92
V05-5973W × V09-3876	16	1	4	25	1	4	25	1	3	33
V05-5973W × V09-3984	13	2	4	50	2	4	50	3	6	50
Total	86	20	26	77	20	26	77	27	42	64

^a^NC: number of lines correctly selected as low SCD by marker

^b^NS: number of lines selected as low SCD by marker

## Discussion

The quality of natto is largely cultivar dependent. Given its ramifications for consumer acceptance and production costs, developing natto soybean cultivars with lower incidence of SCD is of considerable interest to the natto industry. However, screening lines for SCD soybean is time-consuming and an effective selection tool is needed. In an effort to identify QTL and markers associated with low SCD, two sets of biparental populations and their parents were evaluated for SCD, QTLs associated with SCD were identified on Chr. 15 and 20. SNP markers derived from the two stable QTLs were developed and validated to select low-SCD natto soybean.

Large SCD variance was observed among individuals in Pop 1 (0–94%) and Pop 2 (2–96%), consistent with previously reported SCD variation (Cook and Rainey 2010; Yasui et al. 2017). Significant variation in SCD was observed among different years in both populations. SCD was much more severe in 2017 than in 2016 and 2018 for both populations, suggesting that expression of this trait may be particularly influenced by environmental conditions (Table 1). Interestingly, the average temperature and total precipitation during pod development stage (August and September) were much lower in 2017 than in 2016 and 2018 (Table 3S). Low temperature can directly jeopardize soybean seed quality, as chilling temperatures during flowering and low moisture content are both conducive to seed coat cracking (Koizumi et al. 2008). Despite the differences in environmental conditions, continuous distributions were still observed in both populations in individual and across years. This suggests that while environmental influence holds potential to exacerbate absolute SCD incidence, recovery of genotypes that show consistently lower relative SCD is possible through targeted breeding.

Although SCD is widely recognized as an important trait for natto cultivar development, few studies have investigated its genetic control. To address this need, we utilized two biparental mapping populations and identified seventeen QTLs associated with SCD (Table 2). Two stable QTLs were detected across multiple years and populations. A similar QTL study was conducted to study seed coat cracking during natto producing processes (soaking and cooking) in 126 recombinant inbred lines (Yasui et al. 2017). They found that QTL regions on Chr. 4, 6, and 8 were likely responsible for seed coat cracking; however no stable QTL was identified. The reliability of QTL mapping depends on the phenotyping methods, population size, linkage map density, and diverse environment factors. In order to increase the likelihood of identifying stable QTLs, we evaluated larger mapping populations over 3 years (environments) while observing greater marker density per chromosome. Thus, the QTLs identified in this study hold greater potential for future studies regarding the molecular mechanism and genetic basis of SCD in soybean.

The QTL *qSCD15,* flanked by Gm15_5312718 and Gm15_6272006, included two significant SNPs. Gm15_5312718_C_T and Gm15_6272006_T_C were located at 5,331,364 bp and 6,291,081 bp (respectively) on Chr. 15 of the Wm82.a2.v1 reference genome. Even though no QTL associated with soybean seed traits was reported within this region, a previous study identified nearby QTLs associated with soybean seed coat hardness (Kuroda et al. 2013). Though further correlation studies must be conducted, the evidence from both our study and the study by Kuroda et al. (2013) suggest that seed coat deficiency and seed coat hardness might be closely related and that Chr. 15 may be particularly important for modification of seed coat traits in soybean.

Nine significant SNPs were identified with the *qSCD20* region. This region was flanked by Gm20_34881595 and Gm20_36095037, ranging from 36,021,058 bp to 37,190,252 bp on Chr. 20. QTLs responsible for seed composition (oil and isoflavones), seed set, and hilum color have been previously reported in this region (Fang et al. 2017; Leamy et al. 2017; Meng et al. 2016), all of which are also important for natto soybean breeding selection (Escamilla et al. 2019). Similar to the Chr. 15 QTL, *qSCD20* overlapped with a previously identified QTL on Chr. 20 associated with seed coat hardness (Kuroda et al. 2013). Interestingly, *qSCD20* also happened to be located approximately 20 Mbp (12.6 cM based on GmConsensus40) away from another previously reported QTL on Chr. 20 associated with seed coat cracking after harvest (Ha et al. 2012). Seed coat cracking is directly related to the strength of the seed coat. Chr. 20 may harbor several genes associated with seed coat strength and hardness, which play an important role or interact with other genes to cause a complex trait as seed coat deficiency.

We identified candidate genes in the intervals that *qSCD20* and *qSCD15* map using public data on soybase.org. We found 52 genes in the *qSCD20* interval and 95 genes in the *qSCD15* interval. Among those genes in the *qSCD20* interval, we identified one candidate gene, Glyma.20G128600, which is homologous to Arabidopsis CAD4 (AT3G19450.1). This gene is a GroES-like zinc-binding alcohol dehydrogenase family protein involved in lignin biosynthesis. Given that lignin is a key component of the cell wall, it is plausible that alleles governing improved lignin biosynthesis may result in decreased incidence of SCD. We also identified additional candidate genes Glyma.15G078300, Glyma.15G075300, Glyma.15G074700, Glyma.15G074000, and Glyma.15G072300, which are involved in cell wall organization, callose deposition in cell wall, cuticle development, cell wall modification, and secondary cell wall biogenesis (Shao et al. 2007). These candidate genes are targets for future experimental validations of the molecular mechanisms of SCD in soybean. Gene lists underlying both QTLs are provided as a supplementary table with the candidate genes highlighted (Table 4S).

The broad-sense heritability (*H*^2^*)* of SCD in Pop 1 was 0.67, which was also observed for seed coat cracking after soaking (Yasui et al. 2017). Meanwhile, the broad-sense heritability of SCD was higher in Pop 2 (0.83). The reported heritability of other food-grade seed traits such as seed size, protein, sucrose, raffinose, and stachyose concentration range from 0.45 to 0.86 in soybean (Jaureguy et al. 2011). According to Robinson et al. (1949), trait heritability is categorized in three levels: low (0–30%), medium (31–60%), and high (> 60%). Heritability estimates indicate the potential to achieve genetic gain for a trait through breeding selection (Jaureguy et al. 2011). The high broad-sense heritability and large variance in SCD incidence observed in this study suggest that the reduction in SCD in natto soybean cultivars by accumulating favorable alleles through breeding selection is promising. Moreover, the QTLs identified in this study can be used to increase efficiency of breeding selection for the low-SCD trait, which can ultimately permit the development of superior natto soybean cultivars.

Eleven SNP markers were developed from the two stable QTLs located on Chr. 15 and 20 and validated in four diverse biparental populations. None of the markers from Chr. 15 were polymorphic between the parental lines and thus were not suitable for marker-assisted selection in the four populations. Three markers located on Chr. 20 were polymorphic between the parental lines and segregated in the breeding lines of validation populations. Markers Gm20_34626867 and Gm20_34942502 showed the same selection efficiency pattern in the validation populations and both showed higher selection efficiency in populations derived from the low-SCD (2%) parent MFS-561 and relatively lower selection efficiency in populations derived from low-SCD (15%) parent V05-5973W. Marker Gm20_35625615 showed relatively higher selection efficiency in populations derived from MFS-561 than that in populations developed from V05-5973W, which was consistent with the other two markers. The pedigree of MFS-561 shows that its parent MFS-553 is the grand parent of V16-1626 and V12-1885 used for QTL mapping (Fig. 2S). V05-5973W doesn’t share any parents traced back to two generations with V16-1626 and V12-1885. Therefore, the markers explored in this study would be ideally used for SCD selection on populations derived from MFS-553. In addition, the different selection efficiency across populations might indicate the variation of recombination rate between the test markers and low-SCD genes, or the possible involvement or interaction with other QTL. The overall selection efficiency of three markers was 56% in four populations with higher selection efficiency on MFS-561-derived populations and lower selection efficiency on V05-5973W-derived populations, when single-marker selection efficiency was compared. The results indicated that multiple markers should be employed to make better selection decision when using marker-assisted breeding approach.

In summary, two stable QTLs associated with low SCD were identified on chromosomes 15 and 20 by evaluating two biparental populations in three environments. Furthermore, three markers (Gm20_34626867, Gm20_34942502 and Gm20_35625615) were developed based on the stable QTL located on Chr. 20. The markers had a high selection efficiency in two populations with MFS-561 genetic background. The finding of these two major QTLs will shed light on the genetic control of seed coat deficiency, and the markers developed from this study will facilitate molecular marker-assisted selection of low-SCD natto soybean.

## Electronic supplementary material

Below is the link to the electronic supplementary material.

Supplementary material 1 (DOCX 882 kb)

Supplementary material 2 (DOCX 33 kb)

Supplementary material 3 (DOCX 39 kb)

Supplementary material 4 (XLSX 51 kb)
