# Supplementary material for: Identification and validation of major QTLs associated with low seed coat deficiency of natto soybean seeds (Glycine max L.)
Source: Theor Appl Genet. 2020 Aug 26;133(11):3165–76. doi: 10.1007/s00122-020-03662-5 (PMC7547995; doi:10.1007/s00122-020-03662-5)
Supplement: Supplementary file 1 — Supplementary material 1 (DOCX 882 kb) [file 122_2020_3662_MOESM1_ESM.docx]

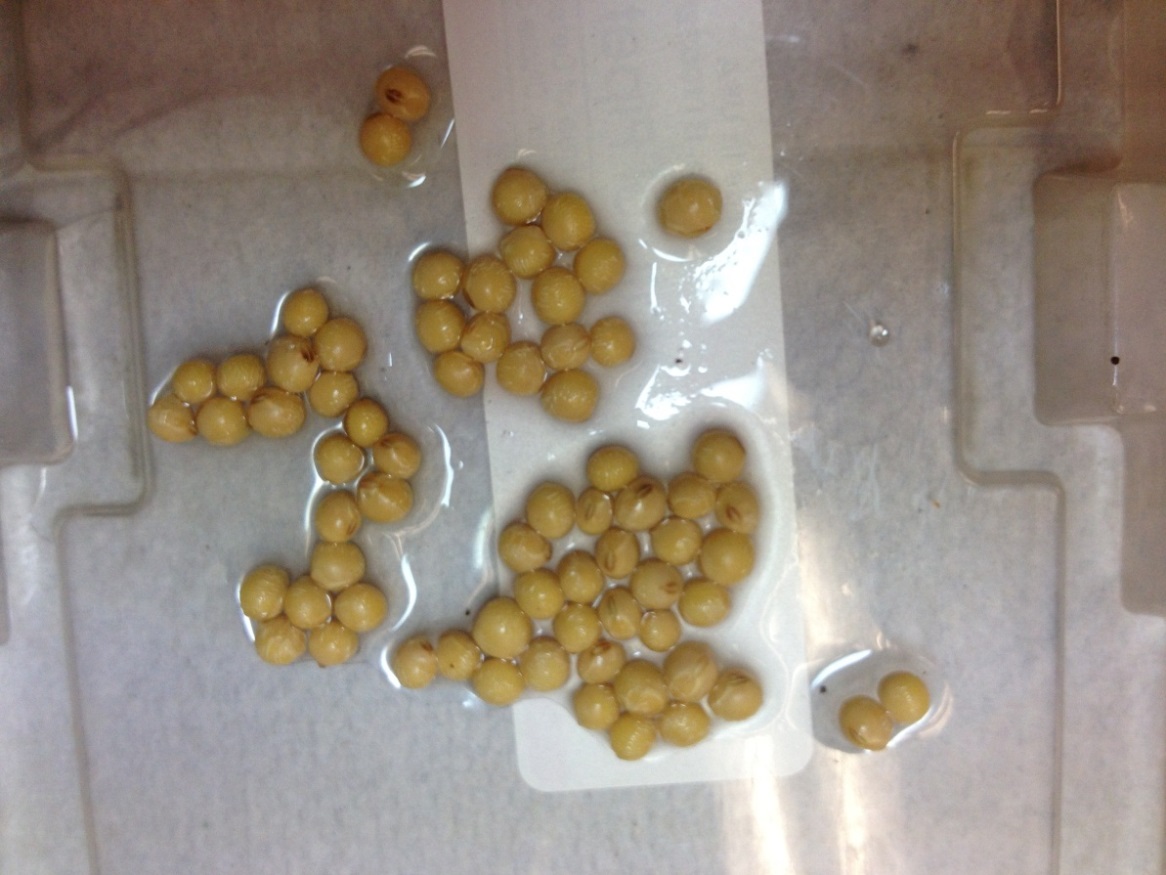

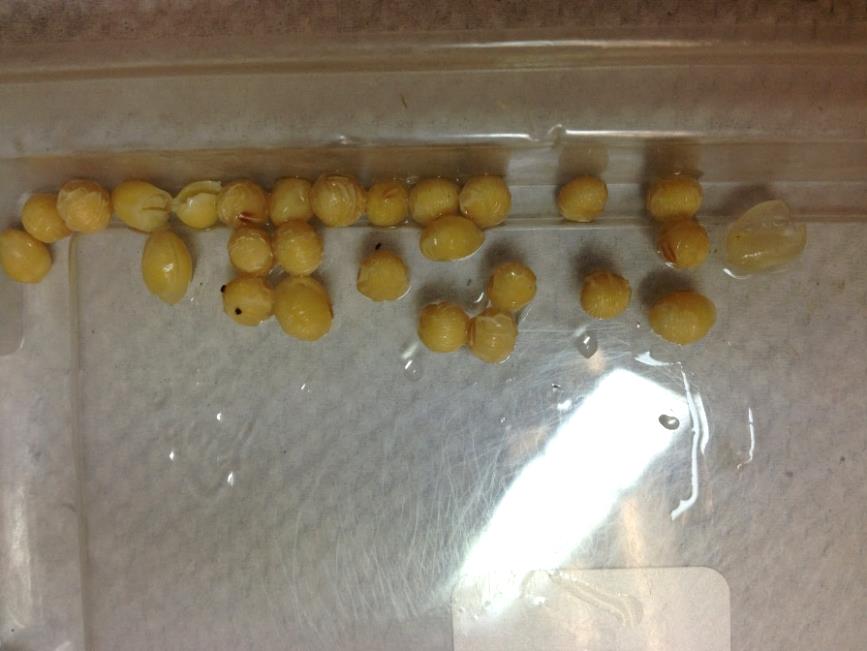


a b

Figure. 1S Soybean seeds show seed coat intact (a) and deficient (b) after soaked in 1% commercial bleach solution for ten minutes.
