# Supplementary material for: Identification and validation of major QTLs associated with low seed coat deficiency of natto soybean seeds (Glycine max L.)
Source: Theor Appl Genet. 2020 Aug 26;133(11):3165–76. doi: 10.1007/s00122-020-03662-5 (PMC7547995; doi:10.1007/s00122-020-03662-5)
Supplement: Supplementary file 2 — Supplementary material 2 (DOCX 33 kb) [file 122_2020_3662_MOESM2_ESM.docx]

V01-2245

V00-3488

G03-3113

R04-198

SS-516

OZARK

V03-0986

DPL 415

MFS-553

DPLX 436

Camp

ESSEX

Holladay

V96-4249

**V12-1626**

**V12-1885**

**V11-0883**

**Figure 1S.** Pedigree of soybean [*Glycine max* (L.) Merr.] lines used in this study. Parental lines used in this study are shown in bold.
