# Supplementary material for: Identification and validation of major QTLs associated with low seed coat deficiency of natto soybean seeds (Glycine max L.)
Source: Theor Appl Genet. 2020 Aug 26;133(11):3165–76. doi: 10.1007/s00122-020-03662-5 (PMC7547995; doi:10.1007/s00122-020-03662-5)
Supplement: Supplementary file 3 — Supplementary material 3 (DOCX 39 kb) [file 122_2020_3662_MOESM3_ESM.docx]

**Table 1S** Summary of single nucleotide polymorphism (SNP) markers used in the population derived from V11-0883×V12-1626

| Chr.^a^ |  | LG^b^ | Length (cM) |  | No. of  selected SNPs | No. of  mapped SNPs | Average distance  between SNP (cM) |
| --- | --- | --- | --- | --- | --- | --- | --- |
| 1 |  | D1a | 91.4 |  | 69 | 68 | 1.3 |
| 2 |  | D1b | 82.2 |  | 45 | 42 | 2.0 |
| 3 |  | N | 128.6 |  | 79 | 78 | 1.7 |
| 4 |  | C1 | 101.2 |  | 49 | 49 | 2.1 |
| 5 |  | A1 | 98.3 |  | 81 | 81 | 1.2 |
| 6 |  | C2 | 79.5 |  | 44 | 43 | 1.9 |
| 7 |  | M | 87.6 |  | 52 | 52 | 1.7 |
| 8 |  | A2 | 108.5 |  | 54 | 54 | 2.0 |
| 9 |  | K | 79.1 |  | 49 | 32 | 2.5 |
| 10 |  | O | 102.5 |  | 58 | 57 | 1.8 |
| 11 |  | B1 | 98.2 |  | 55 | 55 | 1.8 |
| 12 |  | H | 47.7 |  | 33 | 20 | 2.4 |
| 13 |  | F | 97.7 |  | 105 | 104 | 0.9 |
| 14 |  | B2 | 95.8 |  | 110 | 109 | 0.9 |
| 15 |  | E | 78.1 |  | 68 | 68 | 1.1 |
| 16 |  | J | 44.9 |  | 31 | 30 | 1.5 |
| 17 |  | D2 | 87.8 |  | 74 | 63 | 1.4 |
| 18 |  | G | 86.8 |  | 117 | 108 | 0.8 |
| 19 |  | L | 118.0 |  | 85 | 85 | 1.4 |
| 20 |  | I | 112.6 |  | 60 | 60 | 1.9 |
| Mean |  |  | 91.3 |  | 65.9 | 62.9 | 1.6 |
| Total |  |  | 1,826.5 |  | 1,318 | 1,258 |  |

^a^Chr.: chromosome

^b^LG: linkage group

**Table 2S** Summary of single nucleotide polymorphism (SNP) markers used in the population derived from V11-0883×V12-1885

| Chr.^a^ |  | LG^b^ | Length (cM) |  | No. of  selected SNPs | No. of  mapped SNPs | Average distance  between SNP (cM) |
| --- | --- | --- | --- | --- | --- | --- | --- |
| 1 |  | D1a | 62.2 |  | 58 | 58 | 1.1 |
| 2 |  | D1b | 46.5 |  | 49 | 48 | 1.0 |
| 3 |  | N | 55.9 |  | 85 | 83 | 0.7 |
| 4 |  | C1 | 85.2 |  | 102 | 99 | 0.9 |
| 5 |  | A1 | 71.4 |  | 69 | 68 | 1.1 |
| 6 |  | C2 | 59.4 |  | 98 | 97 | 0.6 |
| 7 |  | M | 68.0 |  | 41 | 41 | 1.7 |
| 8 |  | A2 | 73.9 |  | 93 | 93 | 0.8 |
| 9 |  | K | 50.4 |  | 71 | 71 | 0.7 |
| 10 |  | O | 90.5 |  | 92 | 91 | 1.0 |
| 11 |  | B1 | 48.6 |  | 54 | 54 | 0.9 |
| 12 |  | H | 39.2 |  | 49 | 47 | 0.8 |
| 13 |  | F | 59.5 |  | 125 | 122 | 0.5 |
| 14 |  | B2 | 43.2 |  | 126 | 123 | 0.4 |
| 15 |  | E | 46.7 |  | 92 | 92 | 0.5 |
| 16 |  | J | 33.2 |  | 87 | 83 | 0.4 |
| 17 |  | D2 | 71.7 |  | 86 | 78 | 0.9 |
| 18 |  | G | 42.5 |  | 99 | 96 | 0.4 |
| 19 |  | L | 82.8 |  | 105 | 104 | 0.8 |
| 20 |  | I | 59.3 |  | 56 | 56 | 1.1 |
| Mean |  |  | 59.5 |  | 81.9 | 80.2 | 0.8 |
| Total |  |  | 1,189.8 |  | 1,637 | 1,604 |  |

^a^Chr.: chromosome

^b^LG: linkage group

**Table 3S** Weather condition during soybean pod formation period in 2016, 2017 and 2018 in Blacksburg, VA

| Month | 2016 | | 2017 | | 2018 | |
| --- | --- | --- | --- | --- | --- | --- |
|  | T_m_ (℃)^a^ | WTR (mm) ^b^ | T_m_ (℃)^a^ | WTR (mm) ^b^ | T_m_ (℃)^a^ | WTR (mm) ^b^ |
| July | 24 | 123 | 23 | 84 | 22 | 60 |
| August | 23 | 115 | 21 | 64 | 22 | 138 |
| September | 21 | 170 | 18 | 37 | 21 | 200 |
| October | 14 | 64 | 14 | 196 | 13 | 123 |

^a^T_m_: average temperature through the month

^b^WTR: Total precipitation for the month
